# Behavioral, haematological, and physiological effects of oxygen‐rich spray after simulated catch and release in rainbow trout (*Oncorhynchus mykiss*)

**DOI:** 10.1111/jfb.16003

**Published:** 2024-11-30

**Authors:** Nuria Ruiz, Lluis Tort

**Affiliations:** ^1^ Department of Cell Biology, Physiology and Immunology Universitat Autonoma de Barcelona Bellaterra Spain

**Keywords:** angling stress, behavior, delayed mortality, oxygenated spray, reflex impairment

## Abstract

This study investigated the impact of an oxygen‐rich spray on rainbow trout subjected to short‐term air exposure episodes. These episodes can be due to sampling procedures or catch‐and‐release (C&R) practices, focusing on behavioral, haematological, and physiological responses. For this, 12 rainbow trout were divided into two groups: control and oxygen‐rich sprayed fish. The fish were chased for 3 min and exposed to air for 6 s, and blood and behavior parameters were assessed. Simulated C&R resulted in partial physiological reflex impairment across groups. However, sprayed fish exhibited significant differences during recovery in head complex reflex and faster tendencies to regain orientation compared to control. Haematological analyses revealed that the treated group displayed reduced erythrocyte swelling and maintenance of red blood cell (RBC) counts, indicating reduced need for compensatory responses to hypoxia. Meanwhile, stress‐related indicators, including cortisol, glucose, and lactate, remained unaffected by the treatment, suggesting no interference of spraying with the fish ability to launch a healthy stress response. No adverse effects were observed on skin surfaces or gills after an exploratory analysis. Overall, the oxygen‐rich spray exhibited favorable effects, indicating potential benefits in protecting rainbow trout during C&R practices or sampling episodes. Moreover, there is a possible wider application of this methodology to benefit other species of fish subjected to stressors such as aquaculture, research, and fisheries management.

## 1 INTRODUCTION

The importance of fishing extends beyond its food security and economic contributions as it also contributes to human well‐being and social interactions (Arlinghaus et al., 2021). From an anthropocentric view, fishing supporters claim that this activity often provides a serene environment with the opportunities to escape from the stresses of daily life and promote mental health, add substantial amounts of physical exercise, and ultimately connect with nature, fostering an appreciation for the environment and biodiversity (Griffiths et al., 2017). Regardless of the method or location, the intricate interplay between anglers and ecological networks across various scales highlights recreational fishing as a unique social–ecological system (SES) with substantial cultural and social human benefits (Björkvik et al., 2023). It is estimated that 220–700 million people engage in recreational fishing globally (Arlinghaus et al., 2015), and approximately 30 billion fish are caught and released recreationally every year (Cooke & Cowx, 2004), significantly influencing fish population dynamics (Embke et al., 2022).

The catch‐and‐release (C&R) approach to recreational fishing is a practice that involves catching fish, taking an optional picture and/or recording their measures and promptly returning them to their natural habitat (Arlinghaus et al., 2007). The primary objective of release after catch is to minimize harm to the fish and ensure their survival after release. The survival rate in C&R is difficult to measure, especially in long‐term effects (Keefe et al., 2022). It should be added that in a short‐term experiment performed on rainbow trout, the survival was about 96.6% (Pope et al., 2007), and in a long‐term experiment on salmon, the survival was about 92% (Gargan et al., 2015), indicating a mortality range between 0.2 and 1. Thus, C&R contributes to reduce the anglers' impact on fish populations and preserves the welfare of the caught fish, helping to sustain healthy aquatic environments for future fishing endeavors. This method also stands as a more sustainable solution for fisheries management, countering the habitat loss and mitigating the risks of overfishing that typically accompany commercial fishing practices (Arlinghaus et al., 2021; Björkvik et al., 2023).

Although C&R is acknowledged as a regulation for harvesting (size limits and harvest bans), a management technique, or a philosophy in angling, its perception varies across different regions of the world and among distinct groups of anglers (Aas et al., 2002). Despite its intent to ensure the survival of released fish, mortality rates associated with C&R vary among fish species, influenced by many factors (Muoneke & Childress, 1994). These not only include the angling practices (bait type, hook/lure type, body size, and anatomical location of the injury) but are also significantly affected by the environment (water temperature, depth, and dissolved oxygen) (Gale et al., 2013) and the angler behavior during hooking, playing, landing, and/or handling the fish (Danylchuk et al., 2018; Ferter et al., 2013). In the past two decades, a significant body of evidence was collected in an attempt to understand immediate and delayed mortality associated with C&R (Bartholomew & Bohnsack, 2005; Cooke & Suski, 2005; Meka & McCormick, 2005; Pollock & Pine III, 2007; Skov et al., 2023; Stone, 2020), and to develop improved fishing regulations to support both recreational fishing and the environment (Ayllón et al., 2019). Long‐term mortality is difficult to assess, and most of the time it is assumed to be zero. Nevertheless, there can be injuries caused by the C&R that induce later mortality (skin abrasion, mouth injuries, and haemorrhages) (Pollock & Pine III, 2007). Also, in a long‐term study conducted in New Zealand (Dedual & Rohan, 2016), it was observed that the fish abundance decreased after 15 years, so some alteration occurred. Moreover, in another long‐term study lasting for 4 months, the survival rate was about 92% (Gargan et al., 2015). As every fish caught and released into the wild returns to its environment in a diminished state, there is a critical need for innovative and sustainable fishing practices and handling methods to minimize harm and ensure the vitality of aquatic ecosystems (Lewin et al., 2006).

C&R fish are often subjected to physical exhaustion, physiological stress, and air exposure during the handling that may also affect their long‐term, post‐release survival, behavior, migration patterns, disease resistance, and predation (Cooke & Philipp, 2004). It was observed that air exposure time after the C&R practice can affect significantly the physiological and behavioral response of the fish (Arlinghaus et al., 2009). These may be further exacerbated by the global climate change that increases temperature and hypoxia in the aquatic systems (Blaszczak et al., 2023; Jane et al., 2021; Jenny et al., 2016). Rainbow trout (*Oncorhynchus mykiss*) is one of the most popular game species that is fished often under the C&R regulations, and it is highly susceptible to warm waters with low dissolved oxygen (below 5 mgO_2_L^−1^) (Jiang et al., 2021). The average C&R air exposure time for trout was reported as 30 s, and typically under 60 s overall (Lamansky Jr. & Meyer, 2016). Survival of trout is significantly reduced when caught in lower dissolved oxygen conditions or after an air exposure (Ferguson & Tufts, 1992). Other than rainbow trout (Dedual & Rohan, 2016; Lovén Wallerius et al., 2019; Roth et al., 2019), experiments of C&R were also performed in other game fish, including walleye (*Sander vitreus*) (Loomis et al., 2013) and the largemouth bass (*Micropterus salmoides*) (Keretz et al., 2018). Regarding behavior, it was observed that after C&R some fish lost their balance and had to stop to rest, thus increasing the probability of being bitten by a predator (Cooke & Philipp, 2004). Regarding physiological changes, an increase in lactate and glucose and ionic balance levels was observed besides changes in behavior (Arlinghaus et al., 2009). Finally, regarding haematology, sharks under C&R practices showed an increase in haematocrit and haemoglobin (Brill et al., 2008). These data have motivated efforts to refine bass fishing tournaments with the use of live well aerators and water conditioners (Harnish et al., 2011); however, no alternative procedures have been suggested for routine C&R events.

In this study, we used the SwimWell Advanced Fish Recovery Spray developed by ONE with FISH (Denver, CO, USA) that consists of a water‐based, oxygen‐rich formula that can be applied to the fish during the C&R event to support healthy fish physiology while out of water for unhooking or recording the catch. In addition, this spray could be used for other procedures such as sampling episodes in research or aquaculture, where short‐term exposure to air can occur. The primary objective of the study was to assess whether trout treated with SwimWell exhibit an advantageous behavior or physiological responses with respect to the non‐sprayed controls, including the maintenance of the haematological variables and the stress‐associated indicators.

## 2 MATERIALS AND METHODS

### 2.1 Fish husbandry

Twelve rainbow trout (*O. mykiss*), weighing 164.27 ± 41.99 g and measuring 26.94 ± 4.68 cm in length, were transported from the AiguaNatura dels Ports fish farm (Tarragona, Spain) to the AQUAB facility of the Universitat Autonoma de Barcelona (Barcelona, Spain) in February 2023, where they were kept at 18°C (optimal range 5–20°C Molony & Molony, 2001) and in a low density of 5 kg/m^3^ for 3 weeks before experiments began. Fish were housed in 0.25 m^3^ in isolated tanks with a photoperiod 12L:12D and feed at the maintenance ratio of 2% body weight daily with commercial trout pellets (Skretting). Biological and mechanical filters removed suspended solids and maintained ammonia nitrogen levels below 0.002 mg/L. Dissolved oxygen was maintained in the range of 7.5–8 mgO_2_L^−1^, and pH was kept between 7 and 7.5 at all times. All procedures were approved by the Animal Care Committee at UAB and confirmed with the Guide for the Care and Use of Laboratory Animals (protocol number 94 permit OH4218 4219).

### 2.2 The oxygen‐rich spray

**FIGURE 1:**
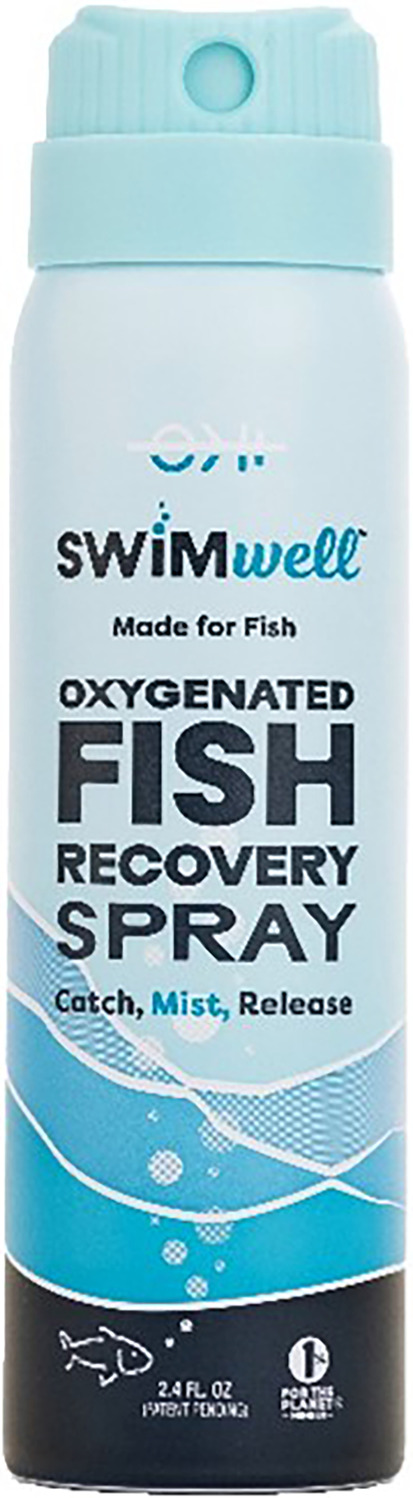
Image of the spray tested. *Source*: One with FISH, Denver, Co.

The SwimWell Advanced Fish Recovery Spray (Figure 1), provided by its manufacturer (ONE with FISH), comes in 2‐oz (59.15‐mL) cans with a twist‐lock actuator and uses bag‐on‐valve barrier packaging pressurized with compressed air. The spray is a water‐based formula containing proprietary‐stabilized oxygen, balanced to physiological ionic strength and pH (Table 1). Its increased viscosity may help the solution stay on the fish's surface and support the fish's mucous slime coat during handling. Dissolved oxygen and pH of the product were quantified using monitor dissolved oxygen test kit based on the Winkler method (JB Chemical and Plastic Co, Bangkok, Thailand) and PH700 benchtop lab pH meter (Apera Instruments, Columbus, OH, USA), respectively, by Much Love Labs (Tulsa, OK, USA).

**TABLE 1:** Parameters of the SwimWell Advanced Fish Recovery Spray.

Formula	Volume, mL	Spray rate, mL/s	Spray time, seconds per side	Fish sprayed, per can	Stabilized oxygen, mg/L	pH, units
SwimWell	59 ± 6	1.0 ± 0.3	1–3	9–27	61 ± 25	7.0 ± 1.6

*Note*: Results are shown as mean ± SD.

### 2.3 Application of the oxygen‐rich spray

All fish (*n* = 12) remained alert when placed into a holding tank and allowed to acclimate to new conditions for a brief exploratory period (10 min). To determine that the fish were acclimated, it was checked that the fish showed a normal swimming behavior (Martins et al., 2012) and an absence of freezing behavior that was described by Vilhunen and Hirvonen (2003). Also, other behaviors that were taken into account to determine that the fish were acclimated were the absence of scape, hiding, and seeking shelter, because those can be related to the flight‐or‐fight response (Sneddon et al., 2016). The stress and exhaustion of the C&R event were then simulated by a 3‐min continuous chase using a dip‐net that was done manually by the observers at a constant speed. Fish swam vigorously in a circular motion to escape from a foreign object. To replicate a successful landing event, fish were held with wet gloves, raised out of the water and into the air, where the control fish (*n* = 6) remained for 6 s. During this time, the treatment group (*n* = 6) was sprayed with the SwimWell Advanced Fish Recovery Spray on both sides for the full length of the body and gills. Spraying kept the surface of the treated fish covered with a water film that remained on the fish for the duration of the air exposure while photography, weighing, and length measures took place. All fish completed the study procedures, and there were no deviations from the study protocol.

### 2.4 Experimental setup

**FIGURE 2:**
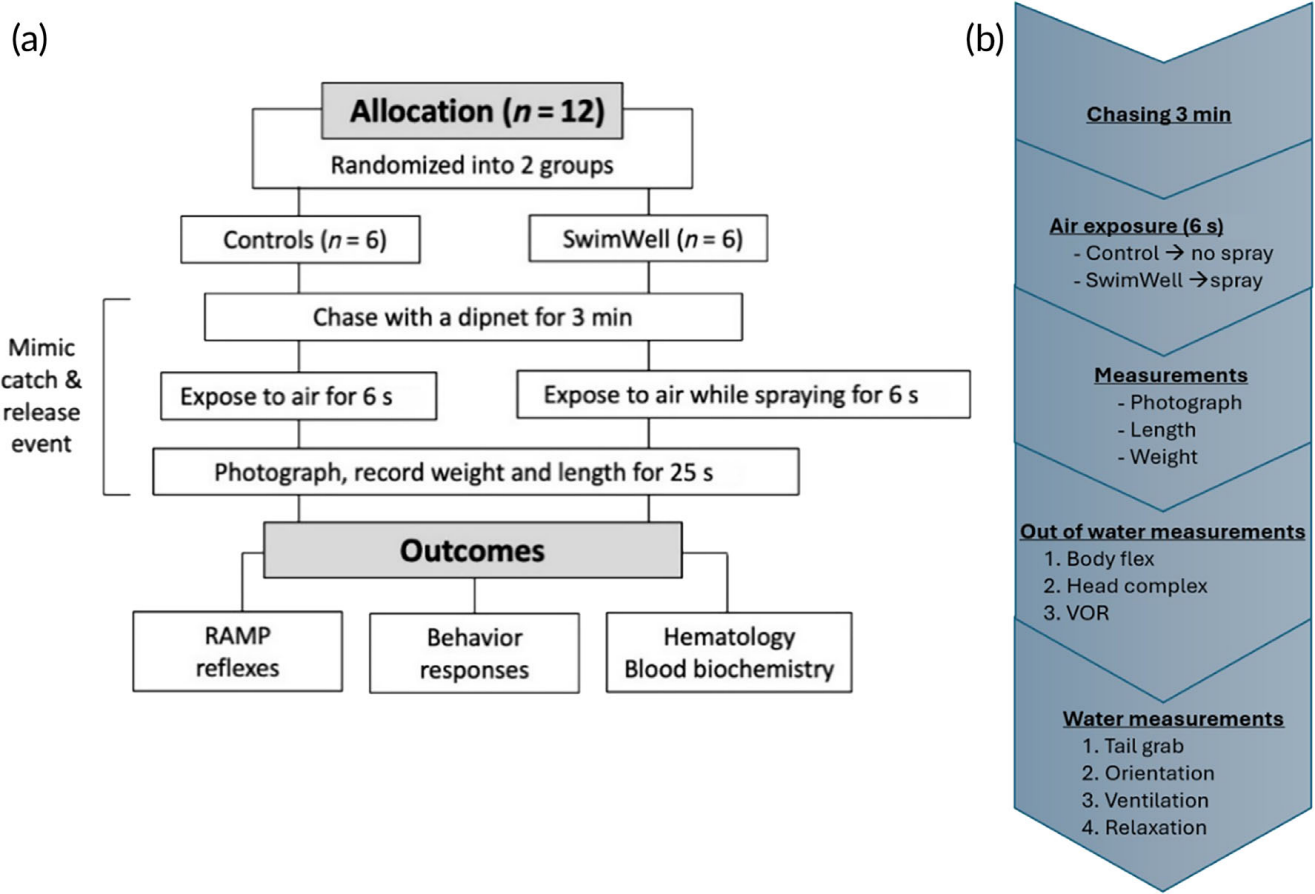
Flowchart of the study. (a) General procedure, (b) Detailed procedure in each step.

All fish (*n* = 12) were randomized into either the control or treatment group (sprayed). Each fish was individually handled to simulate stress and exhaustion associated with a C&R event. After a brief exploratory period, fish were chased with a small dip‐net and swam vigorously in a circular tank for 3 min to mimic the C&R practice. Next, fish were netted and exposed to air for 6 s. During this time, the control fish remained untreated, whereas the treatment group was immediately sprayed with the SwimWell Advanced Fish Recovery Spray for 3 s on each side, misting the entire lengths of the fish body and directly into the gills. The fish was then kept in the air for additional 25 s to photograph, record its weight and length, and blood samples were collected prior to the reflex action mortality predictor (RAMP) evaluations. Fish were then returned to a holding tank for behavioral observations (Figure 2). Apart from these animals, six additional fish were used to establish basal levels for biochemistry parameters.

### 2.5 Physiological reflex assessment

The RAMP scores (Davis, 2010) were used to assess fish condition, with some modifications. The RAMP assessment included a sequential testing of tail grab (escape response), body flex, head complex (ventilation), vestibular‐ocular response (VOR, eye tracking), and orientation (righting response) that was completed in less than 1 min, as described previously (Farrell et al., 2013).

For the evaluation of the head complex reflex, the fish was held out of the water for 5 s while the pattern of regular operculum movements was observed. The fish impairment was scored on a scale from 1 to 3, where 1 represents “regular rhythmic operculum movements,” 2 represents “irregular operculum movements,” and 3 represents “no operculum movements.” For the evaluation of the vestibular‐ocular response reflex, the fish was held out of the water and tilted slowly to one side for 5 s while the counter‐rolling of the eye was observed. The fish impairment was scored on a scale from 1 to 3, where 1 represents “eye rolls steadily,” 2 represents “eye rolls randomly,” and 3 represents “eye does not roll.” For the evaluation of the tail grab reflex, the fish was immersed in the water and attempted to be held at the caudal peduncle. The fish impairment was scored on a scale from 1 to 3, where 1 represents “swims away in a burst,” 2 represents “attempts to swim away but fails,” and 3 represents “does not attempt to escape.”

For the evaluation of the body flex reflex, the fish was held at the middle of the body and lifted out of the water. The fish impairment was scored on a scale from 1 to 3, where 1 represents “flexes frantically,” 2 represents “flexes a few times,” and 3 represents “fails to flex.”

For the evaluation of the orientation reflex, the fish was returned to the water upside down and allowed to recover a proper body orientation. The fish impairment was scored on a scale from 1 to 3, where 1 represents “turns rapidly, 0–20 s,” 2 represents “turns slowly, 20–40 s,” and 3 represents “does not turn effectively, 40–60 s.”

### 2.6 Behavioral recovery

Following the RAMP evaluations, fish were allowed to settle into a steady position in the holding tank. Gill ventilation (the frequency of operculum movements, flaps per minute) and relaxation (time showing the normal exploratory behavior, in seconds). The exploratory behavior was assessed following the criteria previously described (see the review by Martins et al., 2012). For gill ventilation, we counted the time that the operculum was open during 1 min, also for double checking, and to avoid human errors, the count was repeated twice. For relaxation, we monitored the elapsed time that the fish was not moving in the tank until the fish started to show an exploratory behavior and moved a long the tank (Kulczykowska et al., 2012). These parameters were recorded using a video camera, but the analyses were done manually by the trained observers.

### 2.7 Tolerance assessment

At the end of the study after 1 week, all fish were evaluated for signs of damage associated with the experimental procedures, including lesions or abrasions of the skin, bruises and blood marks or haemorrhage, loss of scales, discolouration, and damage to the gills. For this purpose, fish were anaesthetized in a sublethal dose of 100 μg/L ethyl 3‐aminobenzoate methanesulfonate salt (MS‐222) buffered with 200 mg/L of sodium bicarbonate (NaHCO_3_) to do the evaluation of the aforementioned parameters.

### 2.8 Haematology and blood biochemistry

Blood (500 μL) was collected within 3 min after the C&R procedure through caudal puncture using a heparinized syringe. For haematological determinations, 250 μL blood was added to an Eppendorf tube, previously prepared with heparin at the concentration of 1:40 and analysed within 6 h of sampling using the automated flow cytometer blood cell analyser Sysmex XN‐1000 V developed for veterinary analysis and adapted to fish blood cells (Sysmex, Kobe, Japan). Internal quality control was maintained at each daily analysis using three levels of Sysmex reagents (check level 1 or low range, check level 2 or normal range, and check level 3 or abnormally high range). From the data obtained after this analyser, parameters such as the mean corpuscular volume (the relationship between haematocrit and red blood cell count) were calculated.

The second 250 μL blood aliquot was centrifuged at 1500 g for 10 min. The plasma supernatant was immediately separated and frozen at −20°C until analysis of the physiological indicators was performed. Glucose and lactate analyses were performed using colorimetric LO‐POD test kits (Spinreact, Girona, Spain) according to the manufacturer's instructions. For cortisol analysis, the plasma was further diluted in the enzyme‐linked immunosorbent assay (ELISA) buffer, and the aliquots were frozen at −20°C for at least 24 h until quantified with Cortisol ELISA (Neogen, Ayr, UK), as validated previously (Carbajal et al., 2018).

### 2.9 Statistics

Data were analysed using an unpaired two‐tailed *t*‐test with Prism 8.0 (GraphPad Software, San Diego, CA, USA). All data were presented as means ± SD. Significant differences were accepted when the *p*‐value was < 0.05.

## 3 RESULTS

### 3.1 Behavior assessment

**FIGURE 3:**
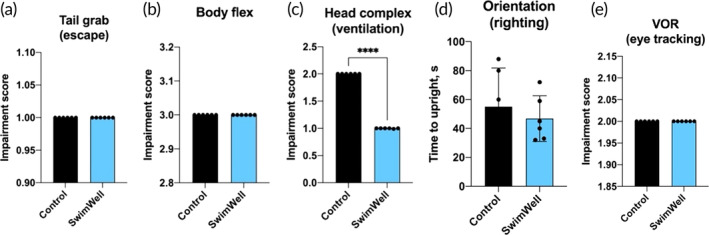
Impairment of the individual physiological reflexes after the simulated catch and release (C&R) event as quantified by the reflex action mortality predictor (RAMP) scores. Reflexes are listed in the order of evaluation, including (a) Tail grab (escape), (b) Body flex, (c) Head complex (ventilation), (d) Orientation (righting reflex), and (e) Vestibular‐ocular response (VOR) (eye tracking). Higher scores indicate more impairment. Values were scored individually and presented as mean ± SD (*n* = 6). Data were analysed using the unpaired two‐tailed *t*‐test, ****p < 0.0001 versus the controls.

Simulating C&R event resulted in a partial loss of the physiological reflexes as evident from the corresponding RAMP scores (Figure 3). The tail grab (escape) and body flex reflex showed no differences between groups in all fish, with no impairment in tail grab in any fish (*p* > 0.05) and a near‐complete loss of the body flex (*p* > 0.05) (Figure 3a,b), as expected after the C&R procedure. The control group also experienced partial impairment of the head complex (ventilation), whereas fish treated with SwimWell showed significant signs of recovery (*p* < 0.0001) (Figure 3c). Sprayed fish also trended toward a faster regain of proper orientation (righting reflex) that did not reach significance (55 ± 11 s in the control vs. 46 ± 6 s in the treated trout, *p* = 0.535) (Figure 3d). Both the control and treated trout showed partial impairment in VOR (eye tracking, *p* > 0.05) reflex that was not affected by the SwimWell treatment (Figure 3e).

### 3.2 Behavioral recovery

**FIGURE 4:**
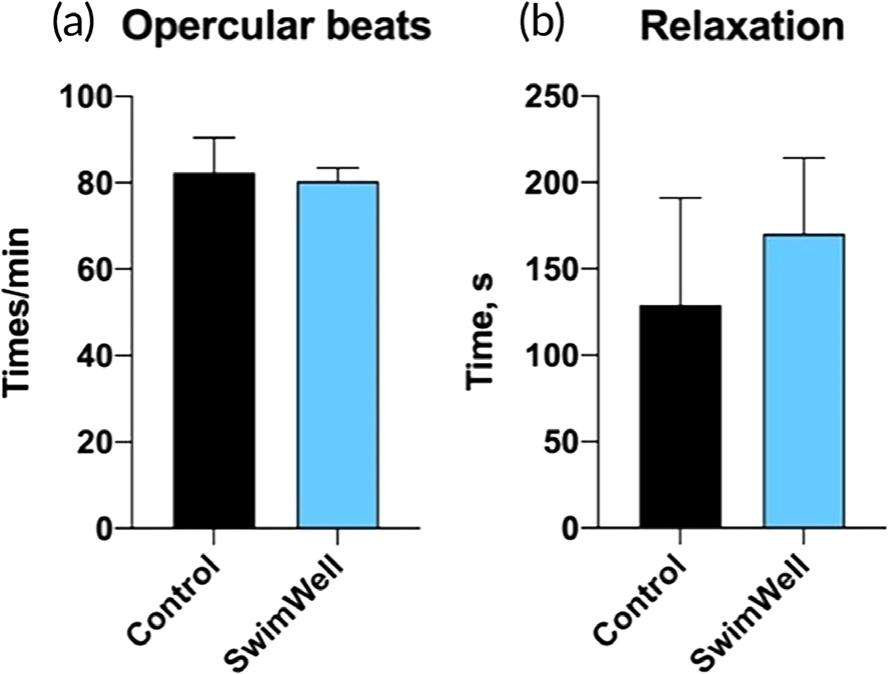
Behavior responses following the simulated catch and release (C&R) event and reflex action mortality predictor (RAMP) evaluation. Fish were returned into the recovery tank and observed for (a) opercular ventilation rates and (b) relaxation behavior. Values were recorded individually and presented as means ± SD (*n* = 6). Data were analysed using the unpaired two‐tailed *t*‐test.

After the evaluation of the RAMP reflexes, fish were returned to the recovery tank and evaluated for their behavior responses that included frequency of ventilation and relaxation (recovery of normal behavior). Ventilation values were similar between the control and treated group (Figure 4a), whereas relaxation time was increased in the SwimWell group but did not show significant differences (170 ± 44 s vs. 129 ± 62 s in controls, *p* = 0.213) (Figure 4b). After the behavior observations were completed, fish treated with SwimWell retained full ability to swim similar to controls (*p* > 0.476) but showed the preference to settle at the bottom of the tank.

### 3.3 Changes in haematological parameters

**FIGURE 5:**
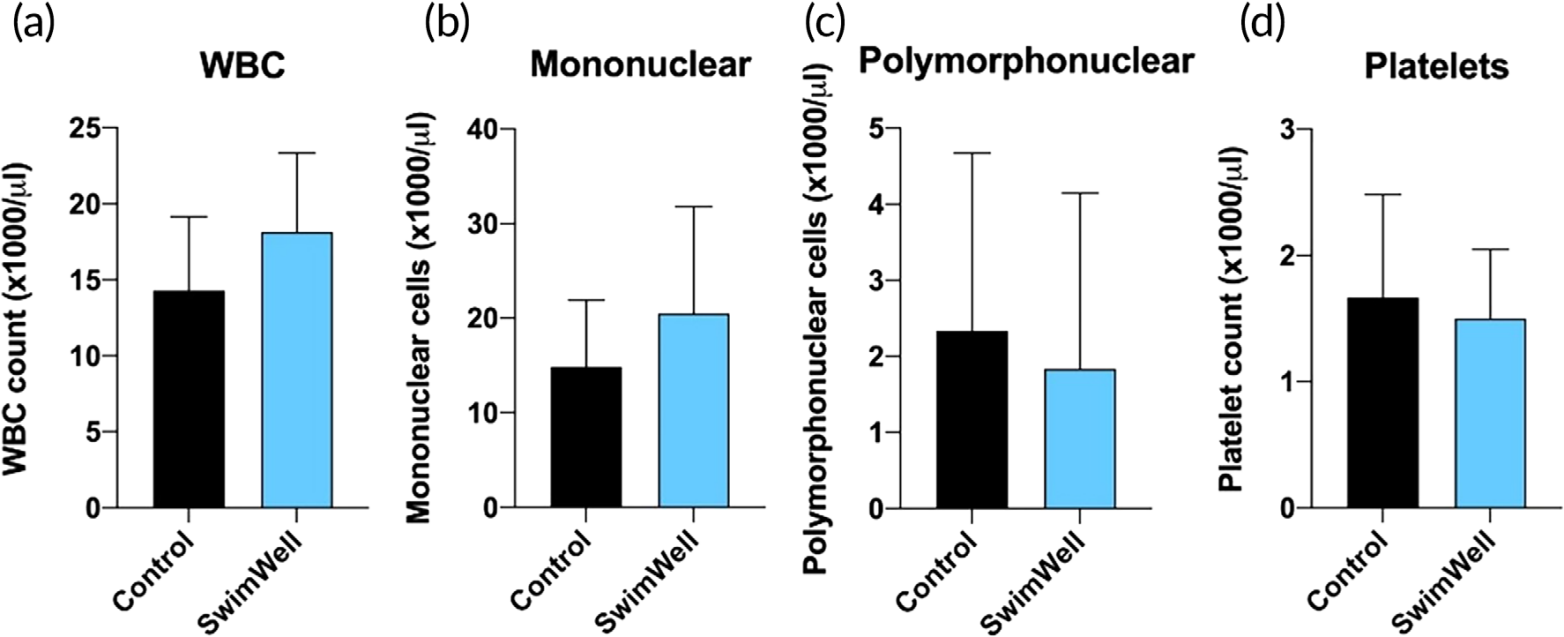
Haematology profiles of (a) Total white blood cells (WBC), (b) Mononuclear leukocytes, (c) Polymorphonuclear leukocytes, and (d) Platelets. Results are expressed as mean ± SD (*n* = 6). Data were analysed using the unpaired two‐tailed *t*‐test.

There were no significant effects on white blood cells (WBCs) and platelet counts (*p* > 0.05) associated with the SwimWell treatment (Figure 5a,d). No differences were also observed in the proportion of mononuclear WBCs that include lymphocytes, monocytes, macrophages, and dendritic cells (*p* > 0.05) (Figure 5b), as well as polymorphonuclear cells that comprise neutrophils, eosinophils, basophils, and mast cells (Figure 5c).

**FIGURE 6:**
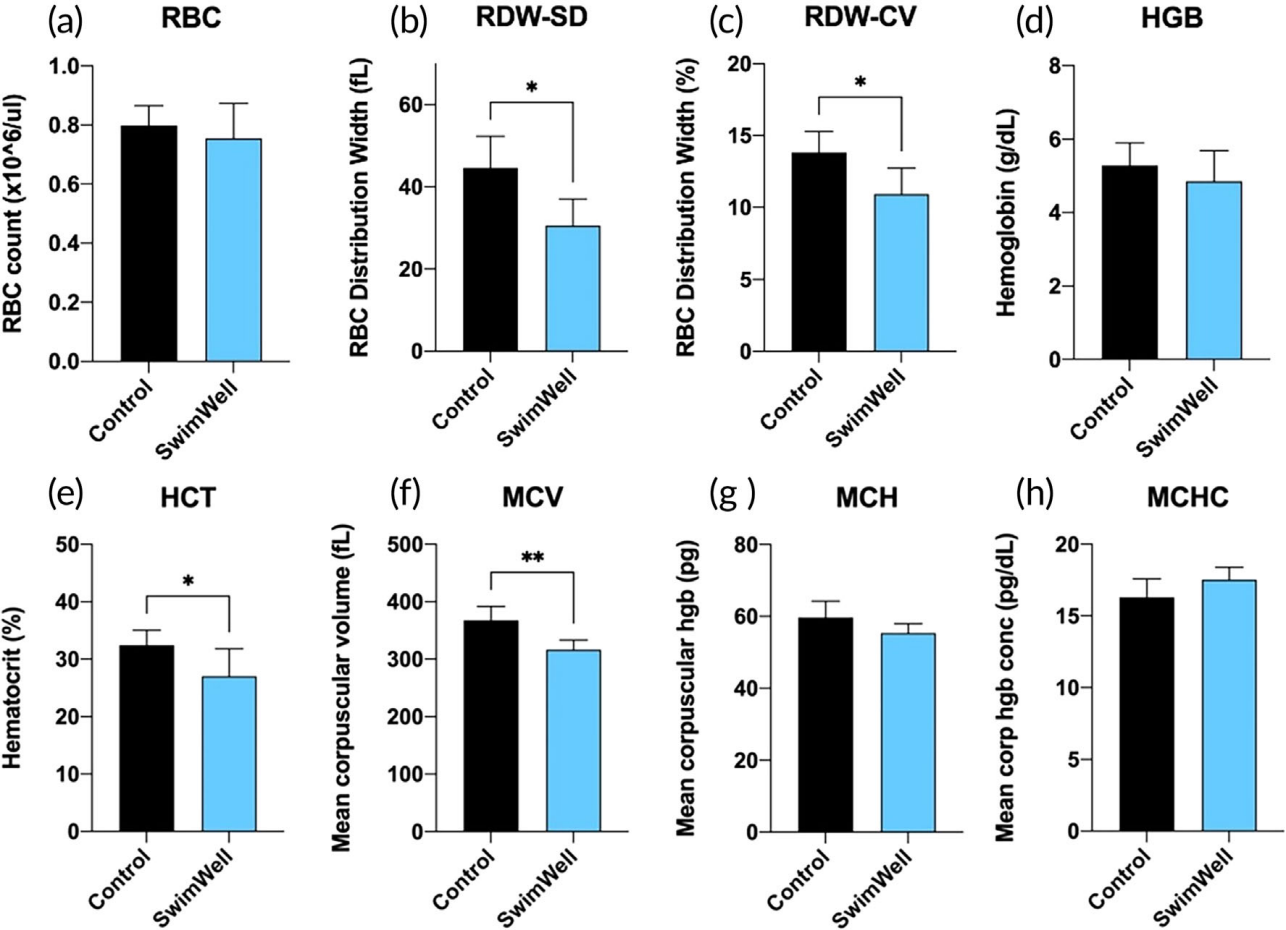
Haematology profiles of (a) red blood cells (RBC), including (b) RBC distribution width (RDW‐SD), (c) RBC distribution width % (RDW‐CV), (d) haemoglobin (HGB), (e) haematocrit (HCT), (f) mean corpuscular volume (MCV), (g) mean corpuscular haemoglobin (MCH), and (h) mean corpuscular haemoglobin concentration (MCHC). Results are expressed as means ± SD (*n* = 6). Data were analysed using the unpaired two‐tailed *t*‐test, *p < 0.05, **p < 0.01 versus the controls.

Application of SwimWell formula to the surface and gills of the trout resulted in several significant changes in the red blood cells count (RBC) parameters (Figure 6). After the simulated C&R event, the total RBC counts remained the same (*p* > 0.05) between the control and treatment groups (Figure 6a), but the control fish showed a significantly increased RBC volume compared to the treated fish, both when analysing the widening of the distribution of red blood cells (RDW‐SD) values (Figure 6b) and the relative RDW‐CV ratio of the RDW‐SD values to the mean RBC volume (*p* = 0.014 and *p* = 0.025, respectively) (Figure 6c). No differences were observed between the control and treated groups in haemoglobin (*p* > 0.05) (Figure 6d); however control fish were observed with significantly higher haematocrit, a percentage by volume of RBC in the blood (Figure 6e). As a result, the mean corpuscular volume was also significantly (*p* > 0.001) increased in control trout, and these effects were alleviated by the SwimWell treatment (Figure 6f). Both mean corpuscular haemoglobin (MCH) (Figure 6g) and mean corpuscular haemoglobin concentration (MCHC) (Figure 6h) were not affected by the SwimWell treatment (*p* > 0.05).

### 3.4 Blood biochemistry and appearance

**FIGURE 7:**
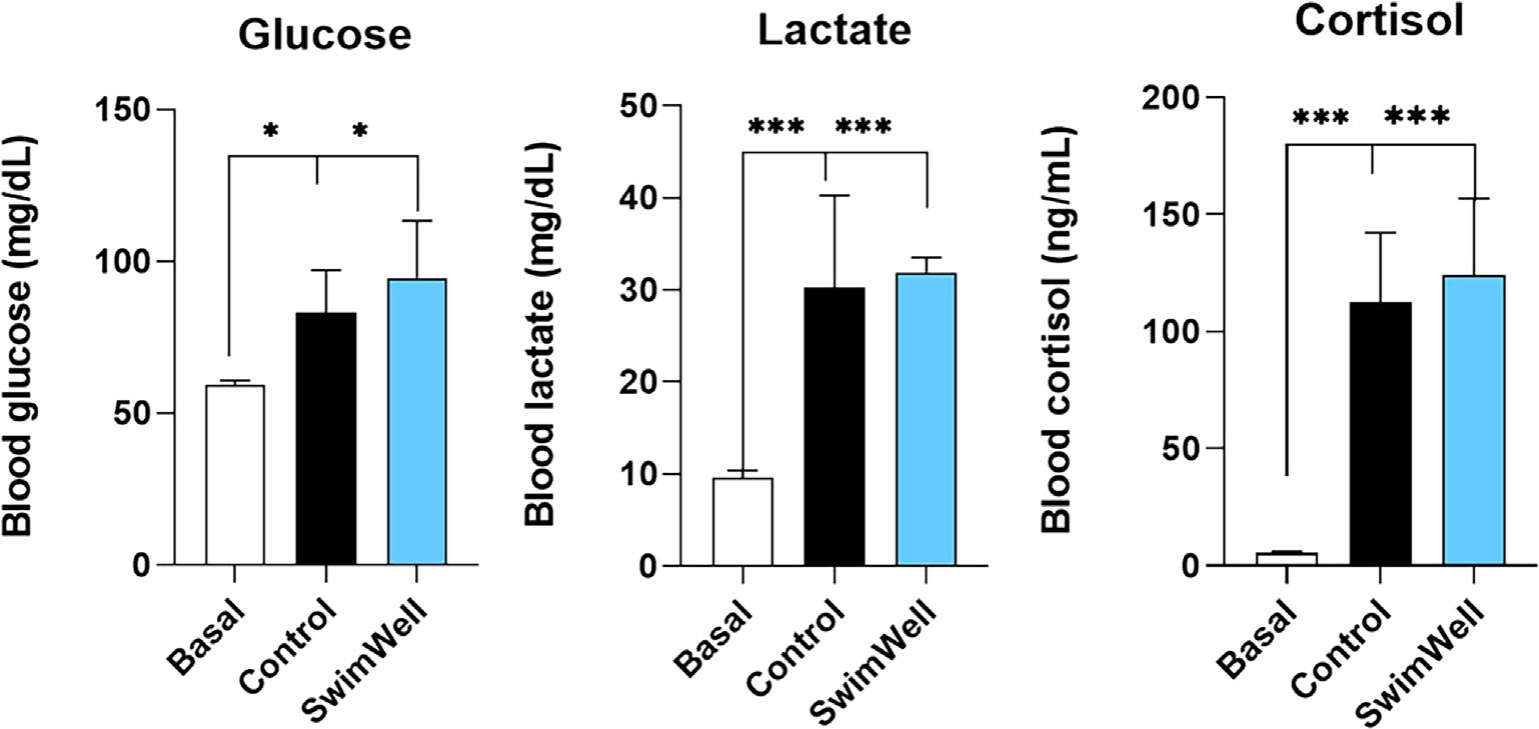
Blood biochemistry profiles including (a) glucose, (b) lactate, and (c) cortisol. Results are expressed as means ± SD (*n* = 6). Data were analysed using the unpaired two‐tailed *t*‐test. Basal values have been added as white bars.

Blood glucose, lactate, and cortisol concentrations were not significantly (*p* = 0.262, *p* = 0.705, and *p* = 0.615, respectively) different between the treatments (Figure 7). There were no significant correlations among these parameters and RAMP scores, and no mortalities occurred in either group. No adverse effects attributable to the intervention were recorded. There were also no significant observations in any group regarding the fish surface, skin, and gill areas suggesting that all fish experienced no negative consequences from the spray application during the study period.

## 4 DISCUSSION

The emergence of C&R represents a paradigm shift in angling approach that reflects a growing appreciation for aquatic ecosystems and the recognition that fishing can coexist with conservation efforts. Also, this work was focused on North America C&R because of available information, but no major differences exist between North America and Britain, for instance, because regulations and the whole business organization associated with C&R are similar. Adoption of C&R strategies is largely driven by two major factors: ethical considerations and a desire to preserve fish populations by the individual anglers, and the mandated management measures that regulate size limits and harvest time (Adams, 2017). In North America, C&R is increasing continuously, as fisheries agencies are promoting initiatives for the 3 R's: recruit new anglers, retain existing ones, and re‐engage those who have stopped (Rempel et al., 2024). The average C&R mortality, however, was estimated at 18% (Bartholomew & Bohnsack, 2005), suggesting that C&R continues to have a significant impact on the environment, even when C&R best practices are being followed (Danylchuk et al., 2018). Trout is typically found on the higher end of the mortality rates up to 32% (Schisler & Bergersen, 1996). Trout mortality is further exacerbated by lower dissolved oxygen (5 mg/L) in water (Jiang et al., 2021) and air exposure (Ferguson & Tufts, 1992). Although most oxygen uptake in fishes occurs across the gills, a substantial amount (6%–30%) of oxygen exchange is also achieved through the skin to support local metabolism, irrespective of scales, due to the presence of a layer of living, non‐keratinized epidermis that covers them (Feder & Burggren, 1985).

Therefore, the aim of this work was to evaluate whether the use of oxygen‐rich spray during a simulated C&R event to keep fish wet during the air exposure protects against hypoxia. This would allow for additional oxygen exchange in the otherwise hostile air environment, and provide physiological advantages over control, untreated fish as measured by the RAMP scores (Davis, 2010), haematological (Caldwell & Hinshaw, 1994), and stress‐related indicators (Schreck & Tort, 2016). RAMP evaluations performed in the sequence of tail grab (escape), body flex, head complex (ventilation), orientation (righting), and VOR (eye tracking) after a simulated C&R event suggested a partial reflex impairment (score = 1) in all categories. Trout sprayed with SwimWell showed significant signs of recovery in head complex and on‐par behavior when tested for other reflexes. The absence of differences between groups in tail grab and in body flex reflex were similar to responses reported in bluegill (*Lepomis macrochirus* Rafin.) and yellow perch (*Perca flavescens* Mitch.) during ice angling (Louison et al., 2017), as well as mahseer (*Tor khudree* Sykes) during the recreational C&R (Bower et al., 2019).

The effect of the oxygen‐rich spray on recovery of the head complex reflex in treated fish, coupled with a trend to regain orientation faster, warrants further investigation. Improved head complex scores indicated that the sprayed fish exhibited rhythmic movements of mouth gape and operculum flare (Farrell et al., 2013) that was impaired in the controls. As fish respiration relies on rhythmic pumping of water across the gills, these findings suggested that fish treated with SwimWell found it easier to obtain the necessary amount of dissolved oxygen to maintain regular breathing and body orientation. If so, it was expected these behavior changes to be associated with the haematological indicators that support this conclusion.

Indeed, under the stress conditions associated with low oxygen exchange, the haematological parameters (Fazio, 2019) may indicate a swelling of the RBC resulting from increased catecholamine release in the control but not in the treated fish. This is evident from a positive correlation between haematocrit (HCT) and mean corpuscular volume (MCV) values (Pavlidis et al., 2007) in the absence of changes in the total numbers of the erythrocytes. In the control fish, this osmoregulatory compensation allowed for increased efficiency of oxygen transfer through the RBC membrane in response to hypoxia‐related stress (Aboagye & Allen, 2018), whereas it was not observed in fish sprayed with SwimWell, presumably due to such osmoregulatory compensation. This haematological response was recorded in the absence of changes in the MCH, suggesting that no hemolysis took place (Swift & Lloyd, 1974). Additionally, the decreased distribution of erythrocytes associated with the heterogeneity and the size of the RBCs (RDW) (Fish et al., 2019) in fish treated with SwimWell supported these findings.

Consistent with simulated C&R stress and air exposure, an increase in blood cortisol, glucose, and lactate was observed in both the control and treated fish, and these biochemical stress indicators (Abdel‐Tawwab et al., 2019) were not affected by the SwimWell treatment. These data indicated that the physiological response (activation of the stress axis leading to cortisol release) to spraying occurred in the absence of direct anaesthetic or attenuating effects associated with the investigated product. Likewise, other downstream indicators of stress, such as glucose and lactate (O'Connor et al., 2011), showed no significant differences between the control and treatment groups in full agreement with the cortisol data. No notable findings were observed on the external surfaces, skin, or gill regions of the fish, implying an absence of adverse occurrences experienced by the fish throughout the study period.

Capturing and handling fish often increase their vulnerability to infections and predators and may cause lasting behavioral changes, including reduced feeding and disrupted migration patterns, impacting their growth, reproduction, and survival. These concerns apply not only to the classical C&R situations but also to the routine procedures in aquaculture (vaccination, biometrics, and routine analysis) or research (population surveys and sampling), where fish also experience handling and short‐term air exposure. Utilizing oxygen‐rich sprays such as SwimWell may enhance the overall condition of released fish, reduce physiological strain, and help fish return to normal behavior more quickly in all these situations. Additionally, oxygen‐rich sprays are ideally positioned to educate anglers and the public about the importance of minimizing harm to fish, develop and promote best practices for fish handling, and explore the broader ecological consequences of their actions.

## 5 CONCLUSIONS

The results of this work indicate that application of the oxygen‐rich spray demonstrated favorable effects in protection against the hypoxia associated with short‐term sampling or simulated C&R practices in rainbow trout. Sprayed fish exhibited an improved head complex reflex and reduced hypoxic haematological indicators, such as erythrocyte swelling and elevated MCV count, implying that the treated group did not develop enhanced compensatory responses in response to stress and exhaustion. The activation of the stress response, as shown by plasma cortisol level, suggested that spraying did not impede the fish's ability to perceive and mount a physiological stress response.

## AUTHOR CONTRIBUTIONS

Conceptualization: Lluis Tort. Methodology: Nuria Ruiz and Lluis Tort. Formal analysis: Nuria Ruiz. Investigation: Nuria Ruiz and Lluis Tort. Resources: Lluis Tort. Data curation: Nuria Ruiz. Writing—original draft preparation: Nuria Ruiz and Lluis Tort. Writing—review and editing: Nuria Ruiz and Lluis Tort. All authors have read and agreed to the published version of the manuscript.

## FUNDING INFORMATION

This research was funded by ONE with FISH (Denver, CO).

## CONFLICT OF INTEREST STATEMENT

The authors declare no conflict of interest.

## INSTITUTIONAL REVIEW BOARD STATEMENT

All procedures were approved by the Animal Care Committee at UAB and conformed with the Guide for the Care and Use of Laboratory Animals Ethics and Animal Care Committee of the “Universitat Autònoma de Barcelona” (permit 94, numbers OH4218 4219 and DAMM 11251), in accordance with the international Guiding Principles for Biomedical Research Involving Animals (EU2010/63).

## Data Availability

Data are contained within the article.
